# REGISTERED REPORTS FOR ECRS: ENABLING SLOW SCIENCE ON TIGHT TIMELINES

**DOI:** 10.1093/geroni/igz038.089

**Published:** 2019-11-08

**Authors:** Rita Ludwig

**Affiliations:** University of Oregon, Eugene, Oregon, United States

## Abstract

Early career researchers (ECRs) may experience tension between the ideal and actual amounts of time they have to complete a scientific project. Sometimes, a timeline is truncated because a citable product is required for applications or fellowship deadlines. This scenario is especially common for researchers who use longitudinal methods, and/or those who work with hard-to-access samples. Registered reports, an open science initiative, offer one resolution to this tension. In registered reports, the steps of analysis planning, manuscript writing, and peer review occur earlier than the traditional journal article publication process. If an in principle acceptance is earned, ECRs are afforded citable, peer-reviewed acknowledgement of their scientific thinking prior to the conclusion of a research project. This talk will serve as a primer on the registered report process. I will also discuss resources for writing registered reports, and provide a list of relevant participating journals in the field of gerontology.

